# Intracranial Pressure Monitoring: Invasive versus Non-Invasive Methods—A Review

**DOI:** 10.1155/2012/950393

**Published:** 2012-06-08

**Authors:** P. H. Raboel, J. Bartek, M. Andresen, B. M. Bellander, B. Romner

**Affiliations:** ^1^Department of Neurosurgery, Copenhagen University Hospital Rigshospitalet, DK-2100, Copenhagen, Denmark; ^2^Department of Neurosurgery, Karolinska University Hospital, SE-17176, Stockholm, Sweden

## Abstract

Monitoring of intracranial pressure (ICP) has been used for decades in the fields of neurosurgery and neurology. There are multiple techniques: invasive as well as noninvasive. This paper aims to provide an overview of the advantages and disadvantages of the most common and well-known methods as well as assess whether noninvasive techniques (transcranial Doppler, tympanic membrane displacement, optic nerve sheath diameter, CT scan/MRI and fundoscopy) can be used as reliable alternatives to the invasive techniques (ventriculostomy and microtransducers). Ventriculostomy is considered the gold standard in terms of accurate measurement of pressure, although microtransducers generally are just as accurate. Both invasive techniques are associated with a minor risk of complications such as hemorrhage and infection. Furthermore, zero drift is a problem with selected microtransducers. The non-invasive techniques are without the invasive methods' risk of complication, but fail to measure ICP accurately enough to be used as routine alternatives to invasive measurement. We conclude that invasive measurement is currently the only option for accurate measurement of ICP.

## 1. Introduction

The Scottish anatomist Alexander Monro first described the intracranial pressure in 1783 [1, 2]. Monro proposed that (1) the brain is encased in a rigid structure; (2) the brain is incompressible; (3) the volume of the blood in the cranial cavity must therefore be constant; (4) a constant drainage of venous blood is necessary to make room for the arterial supply. Monro's colleague George Kellie of Leith supported Monro's observations some years later based on autopsies of humans and animals [3]. These assertions became known as the Monro-Kellie hypothesis or doctrine. However, both were missing a crucial component: the cerebrospinal fluid (CSF). The Flemish anatomist Vesalius had described fluid-filled ventricles back to the sixteenth century, though this view had never been broadly accepted. Not until the French physiologist François Magendie in 1842 through animal experiments punctured the cisterna magna and analyzed the CSF, was the idea of fluid in the brain accepted [4]. 

With this new knowledge in mind, the English physician George Burrows proposed in 1846 the idea of a reciprocal relationship between the volumes of CSF and blood, that is, an increase in one causes a decrease in the other and introduced CSF as a factor in the Monro-Kellie doctrine [5]. 

In 1926, Harvey Cushing, American neurosurgeon, formulated the doctrine as we know it today [6], namely, that with an intact skull, the volume of the brain, blood, and CSF is constant. An increase in one component will cause a decrease in one or both of the other components.

**Figure 1:**
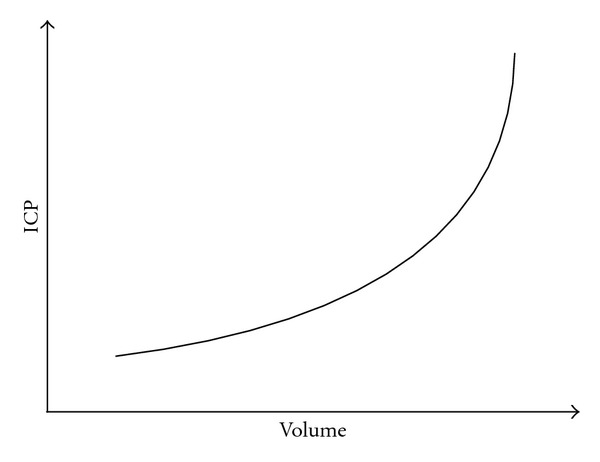
The relationship between intracranial pressure and volume.

This relationship provides a compensatory reserve, also called spatial compensation. It is 60–80 mL in young persons and 100–140 mL in elderly, mainly due to cerebral atrophy [7]. The volume/pressure curve is shown in Figure 1.

The first part of the curve is characterized by a very limited increase in pressure due to the compensatory reserve being large enough to accommodate the extra volume. With increasing volume, the compensatory reserve is eventually exceeded, causing a rapid increase in pressure.

Normal ICP varies with age and body posture but is generally considered to be 5–15 mmHg in healthy supine adults, 3–7 mmHg in children and 1,5–6 mmHg in infants [7–12].

In cases of elevated ICP or circulatory hypotension, the cerebral perfusion pressure (CPP) is decreased. CPP is calculated by subtracting ICP from the mean artery pressure (MAP), defined as the sum of the diastolic pressure added to a third of the difference between systolic and diastolic pressure.

Under normal physiological conditions, the cerebral autoregulation maintains a constant flow of blood to the brain by dilating or constricting the arterioles. However, this autoregulation is only effective with a MAP between 50 and 150 mmHg. Pressure above the upper limit of autoregulation will cause hyperemia and cerebral edema. Pressures below the limit lead to insufficient blood flow and cerebral ischemia, thereby promoting edema formation, which is ultimately associated with a poor patient prognosis. Any lesion of the brain can cause a state of vasomotoric paralysis, where autoregulation is “set out of play,” and cerebral blood flow is entirely dependent on CPP [7, 8, 13–16].

Additionally, elevated ICP can cause herniation with high risk of irreversible brain damage and death [7, 8, 17, 18]. Treatment designed to lower ICP should be initiated at pressures above 15–20 mmHg, depending on the cause of elevated pressure [8, 17].

According to the American Brain Trauma Foundation [19], ICP monitoring is indicated in all cases of traumatic brain injury with a Glasgow Coma Scale score (GCS) between 3–8 and an abnormal CT scan, that is, one showing hematomas, contusion, swelling, herniation, or compressed basal cisterns. Patients with a GCS of 3–8 but a normal CT scan should be monitored if two or more of the following conditions are present: age over 40, uni- or bi-lateral motor posturing, or systolic blood pressure under 90 mmHg.

Causes of elevated ICP are numerous, and ICP monitoring is used in patients with various neurological, neurosurgical, and even medical conditions such as hepatic encephalopathy (Table 1). No universally accepted guidelines exist, and indications for ICP monitoring vary considerably between hospitals [8, 20–22].

ICP monitoring in the pediatric population poses special challenges as well as additional noninvasive techniques of ICP measurement. This will not be addressed further in this paper; however, another useful review is available addressing this topic [23].

Several questions arise when performing ICP monitoring, not only concerning the type of pressure monitor, but also concerning the optimal location of monitoring and where local pressure gradients may influence measurements.

In this context, it is important to address specific conditions, such as communicating or noncommunicating hydrocephalus as well as patients with idiopathic intracranial hypertension, in which there seems to be no transmantle pressure gradient across the ventricular wall [24, 25]. Additionally, in patients with subarachnoid hemorrhage or spontaneous ganglionic hemorrhage, the true intracranial pressure may be estimated by lumbar cerebrospinal fluid pressure [26, 27].

In general, there seems to be a consensus that smaller pressure gradients within the central nervous system do exist across specific compartments, and that they may be exacerbated due to trauma both with and without acute expanding lesions [28–31]. At present, there does not seem to be evidence to support claims of significant pressure gradients under physiological conditions [25, 32].

Early descriptions of pressure gradients [33–36] described differences in pressure between different compartments in the cranial vault as well as along the craniospinal axis. Later reports—as well as experiments in a porcine model—showed compartmentalized ICP pressure gradients [28, 37, 38], indicating that ICP may possibly be best monitored as close as possible to an expanding mass lesion.

To date no studies have conclusively been able to demonstrate how often—and under what circumstances—pressure gradients appear as well as whether bilateral ICP monitoring should be undertaken routinely. However, in the evaluation of trauma patients, the danger of localized elevations of ICP should be taken into account when ICP and clinical symptoms differ markedly.

**Figure 2:**
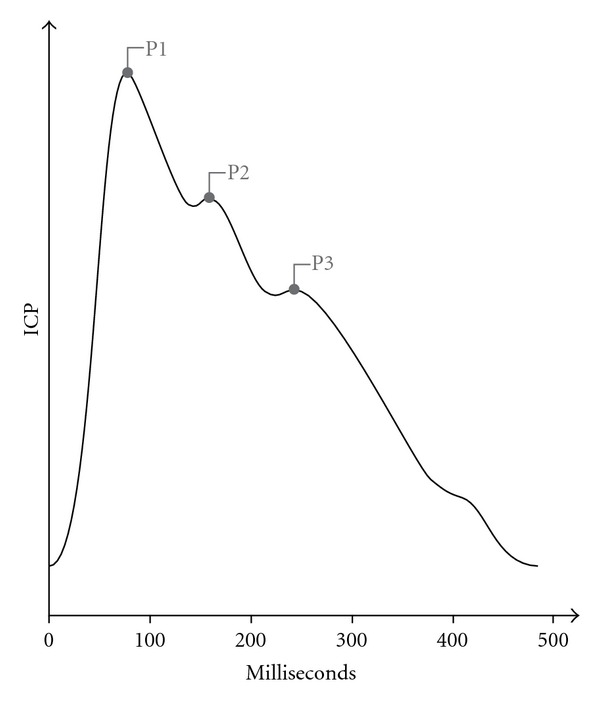
Propagation of the cardiac pulse pressure signal.

Another pitfall in the clinical use of ICP monitoring is in determining the validity of the obtained pressure value. Access to a high-resolution view of the intracranial pressure waveform enables more accurate analysis of the obtained ICP as highlighted by the following examples. Electrostatic discharges may cause both rapid shifts of ICP as well as gradual drifts, which may escape the attention of the clinician [39]. Attention to the mean wave amplitude will show increasing amplitude at increasing ICP, while ICP shifts due to electrostatic discharges will not be accompanied by increasing mean wave amplitude. Furthermore, in performing basic checks of whether the ICP signal is truly representative of the intracranial pressure, the clinician should ensure that there is in fact an oscillating pressure curve with the progressively decreasing P1, P2, and P3 notches present, indicating propagation of the cardiac pulse pressure signal (Figure 2). Further information is found in the pulse pressure signal, with reversal of the P1 and P2 notches reflecting a state of disturbed autoregulation. A complete absence of a pressure curve may additionally be seen following craniectomy and in postoperative pneumencephalon.

More advanced pressure curve analysis may also be employed by identifying Lundberg A- and B-waves [40]. A-waves (also known as plateau waves) are characterized by rapid increase and decrease of pressure to 50–100 mmHg lasting from 5 to 20 minutes. Their duration varies and will often appear irregularly without forewarning. They are a sign of more severe loss of cerebral autoregulation. The rhythmic oscillating B-waves appear with a frequency of 1/2–2 per minute, and may be a sign of cerebral dysfunction, but may in certain cases also be physiological phenomena [41].

## 2. Invasive Methods of Measuring ICP

Several different invasive methods of measuring ICP exist. Depending on the technique, ICP measuring can be undertaken in different intracranial anatomical locations: intraventricular, intraparenchymal, epidural, subdural, and subarachnoidal. Additionally, in patients with communicating CSF pathways, ICP may under certain circumstances be assessed by lumbar puncture [26, 27, 42, 43], as mentioned in the above chapter.

### 2.1. External Ventricular Drainage (EVD)

Invasive monitoring using the EVD technique, where a catheter is placed into one of the ventricles through a burr hole, is considered the gold standard [8, 44–48] of ICP monitoring. In addition to measuring ICP, this technique can also be used for drainage of CSF and administering of medicine intrathecally, for example, antibiotic administration in cases of ventriculitis, possibly resulting from EVD placement itself. During long-term CSF drainage through an EVD, compression of the ventricular system due to progressive edema formation may arise and block proper EVD drainage. Additionally, EVD placement may be indicated to drain posttraumatic hemorrhage.

A word of caution applies for those cases, where a definable, abnormal mass is responsible for intracranial hypertension, and an EVD is inserted for pressure relief. In these cases, caution is advised, since the acute CSF drainage can displace the intracerebral structures, and in severe cases even provoke subinkarceration [49].

Surgical placement of EVD is seen as a minor surgical procedure with few risks, but has nevertheless been associated with hemorrhagic and infectious complications. Regarding the technique and placement for the EVD, traditionally, a coronal burr-hole approach at the Kocher's point, with the tip of the EVD located in the 3rd ventricle is seen as the method of choice, with alternative methods such as the Frazier burr-hole (occipital-parietal), Keen's point (posterior-parietal) and Dandy's point (occipital) as secondary options. Nevertheless, the issue is still under debate, and there has been no general consensus in this field [50].

**Figure 3:**
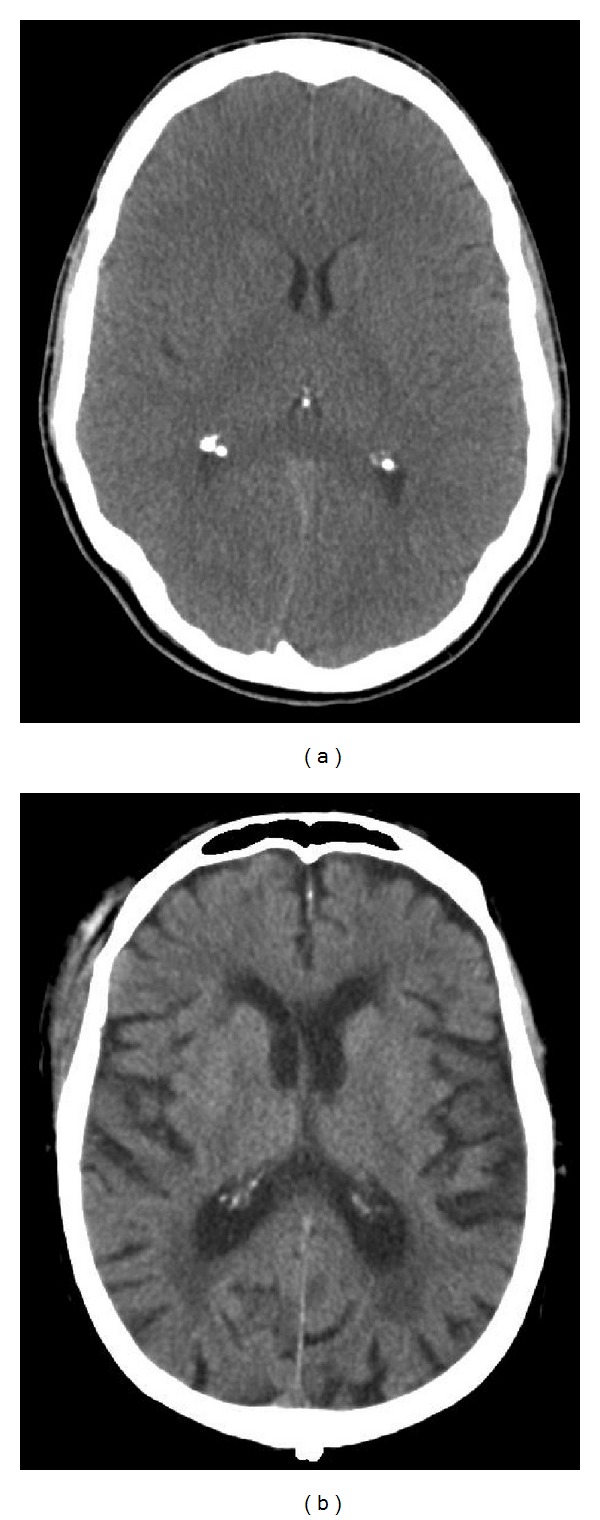
Differences in ventricular size in (a) young and (b) elderly patients.

Depending on ventricular size, EVD placement may be difficult, especially in younger patients with a very slender ventricular system (Figure 3(a)). In the elderly, we often see a widened ventricular system due to age-dependent atrophy, as mentioned earlier (Figure 3(b)).

Focusing on “postoperative hemorrhage,” a review [51] including articles from 1970 and onwards, found hemorrhagic complications in 5,7% of cases on average. This number covers significant differences in incidence, depending on whether a routine CT scan was performed after the procedure. Not surprisingly, hemorrhages were discovered more often in patients who underwent a CT scan (10,06%), than in those who were not routinely scanned after surgery (1.53%). The majority of hemorrhages discovered were of no clinical importance. Of the total amount of hemorrhages, 0,61% were of clinical importance, that is, causing neurological deficits, required surgical intervention, or were fatal.

Another study [52] with a total of 188 patients with EVDs, in which the patients were CT scanned after EVD placement, showed “postoperative hemorrhages” in 41% of cases, though only 10,6% had hemorrhages larger than 15 mL or with an intraventricular component. One patient (0,53%) developed a subdural hematoma requiring surgical drainage.

Another complication in EVD treatment is bacterial colonization of the catheter with subsequent retrograde infection. This encompasses a wide array of conditions, from benign skin infections to ventriculitis, meningitis, and fatal septicaemia.

Review articles on this subject [53, 54] have shown a frequency of catheter-related infections in the range of 0–27%; however, the definition of a catheter-related infection varies tremendously [54]. The majority of reviewed articles used a positive CSF culture obtained from the EVD or drawn via a lumbar puncture [54]. However, it is important to note that a positive CSF culture can stem from other sources, such as cutaneous contamination during the removal process. Factors shown to predispose towards a higher infection rate included; prolonged EVD treatment time of more than five days, frequent CSF sampling rate, intraventricular or subarachnoid hemorrhage, cranial fracture with CSF leakage and nonsterile EVD insertion [54–56]. The primary factor disposing towards a lower infection rate was the subcutaneous tunneling [53].

Minimizing the above predisposing factors, Dasic et al. [57] managed to achieve a significant decrease in the rate of infection, from 27 to 12%, in 95 patients with a total of 113 EVD insertions, by performing the procedure in the sterile environment of a operating theatre, with the use of prophylactic antibiotics, tunneling the catheter subcutaneously at least 10 centimeters from the burr-hole, avoiding routine sampling of CSF (unless clinically indicated) and by not changing the catheter (unless clinically indicated).

Strict adherence to sterile practice is also—according to Tse et al.—the reason for the low rate of infection in another large retrospective study with 328 patients and 368 EVDs [58]. Over 4 years a mean infection rate of 2,98% was reported. They also found, that neither the duration of EVD treatment, surgical revision, urokinase installation, nor preoperative intracranial hemorrhage increased the risk of infection.

Concerning the use of prophylactic antibiotics prior to surgery, Beer et al. [53] argue against this because of the risk of infection with more virulent organisms as well as a theoretically increased resistance to antibiotics. Antibiotic impregnated catheters are an alternative and have proved very effective in reducing the rate of infection [59, 60]; however, they present the same risks concerning resistance [53, 57]. Another option is using catheters impregnated with silver nanoparticles. This technique has good antimicrobial properties *in vitro *[61], but has not been tested thoroughly *in vivo*. A pilot study conducted by Lackner et al. [62] with 19 patients treated with silver nanoparticle impregnated catheters, and 20 controls with regular intraventricular catheters, showed a significant lower incidence of catheter-associates ventriculitis in the test population (zero) than the controls (five). Similar optimistic results were reported from Fichtner et al. [63], that retrospectively reviewed a total of 164 consecutive patients, 90 with a standard EVD and 74 with a silver-bearing EVD, and found a significant reduction in the occurrence of: a positive CSF culture, colonization of the catheter tip, and liquor pleocytosis in the silver-bearing EVD group compared to the group with standard EVD (18,9% versus 33,7%; *P* = 0,04). However, both studies are relatively small, and therefore a larger study with higher statistical power or more centers reproducing the results is needed in order to draw any firm conclusions. 

Another factor contributing to increased infection rate is incorrect placement of the catheter or a defect catheter. Saladino et al. [64] found by retrospectively reviewing a patient population of 138, that 12,3% of all catheters were malplaced either intraparenchymal or extraventricular. This in turn resulted in reoperation in several cases; a factor contributing to higher infection rate. These malplacements can also result in injuries to important cerebral structures, for example, basal ganglia, thalamus, the internal capsule, and even penetration of the floor of the 3rd ventricle.

A so-called “Ghajar guide” has been shown to increase the amount of correctly positioned catheters as compared to insertion by hand [65]; however, according to a survey amongst American neurosurgeons, only 3% use the guide on a regular basis [66]. The rate of defective ventricular catheters has been found to be 6,3%, the cause often being intraparenchymal placement, blockage with pieces of brain matter and blood clots [67–69].

### 2.2. Microtransducer ICP Monitoring Devices

This group of invasive ICP monitoring devices can be divided into fiber optic devices, strain gauge devices, and pneumatic sensors.

Fiber optic devices, such as the Camino ICP Monitor, transmit light via a fiber optic cable towards a displaceable mirror. Changes in ICP will move the mirror, and the differences in intensity of the reflected light are translated to an ICP value. The Codman MicroSensor, the Raumedic Neurovent-P ICP sensor, and the Pressio sensor belong to the group of piezoelectric strain gauge devices. When the transducer is bent because of the ICP, its resistance changes, and an ICP can be calculated. Pneumatic sensors (Spiegelberg) use a small balloon in the distal end of the catheter to register changes in pressure, and additionally allows quantitative measurement of intracranial compliance. Depending on the technique, monitoring can be done in the intraventricular, intraparenchymal, epidural, subdural, or subarachnoidal compartment.

The ICP microtransducers most widely used, are those measuring ICP intraparenchymally, usually placed in the right frontal region at a depth of approximately 2 cm. However, depending on known or suspected pressure gradients across intracranial compartments, the placement can be modified.

Regarding epidural ICP monitoring, currently it does not provide the necessary accuracy for routine use. One report shows that the epidural Camino sensor considerably overestimated ICP with a mean of about 9 mmHg but extending to almost 30 mmHg [70]. Another study showed a markedly differing mean ICP, but comparable parameters of pulsatile ICP (mean wave amplitude and wave rise time) [71].

A comparison between epidurally and subdurally placed pressure sensors [72] showed lower ICP values measured in the subdural space, but obtaining almost equal ICP values in intervals above 20 mmHg. In a newer study comparing lumbar cerebrospinal fluid pressure to epidural and subdural ICP [73], an excellent correlation was found between the lumbar and subdural pressure measurements. However, higher pressure values were consistently found for ICP measured in the epidural space with increasing distance between the lumbar and epidural values at higher pressure intervals. The authors concluded that the higher ICP in the epidural space was due to physiologically different pressures in the two compartments and not due to technical aspects.

For current use in critical care, subdural sensors may be considered for use if there is no suspicion of focal ICP elevations with potential for causing intercompartmental pressure gradients. However, this will seldom be the case, and intraparenchymal or intraventricular monitors should be considered the standard choice. Complications are, as with EVDs, mainly the risk of hemorrhage and infection.

A large study including 1000 patients with a total of 1071 Camino ICP Monitors [74], found retrospectively, that of the 574 probe tips examined, 8,5% were positive for bacterial growth by subsequent cultivation, even though a positive culture growth could stem from cutaneous contamination during the removal process. A control CT scan was undertaken in 92,2% of patient cases and showed an incidence of hemorrhage in 2,5% of cases. In 6 cases (0,66%), a clinically significant hemorrhage was found (4 intraparenchymal and 2 epidural). Technical errors were present in 4,5% of cases, most often related to the fiber optic cable itself. Similar study by Piper et al. [75] found 10% faulty sensors in a study that included a total of 50 Camino monitors. Another study [76], in which 328 patients with a Camino monitor were examined, showed hemorrhage in 1,1% of cases, infection, in 4,75% and technical errors in 3,14%.

The Codman MicroSensor has also been thoroughly examined in several studies; among 120 patients with a Codman MicroSensor, Hong et al. [77] found no incidents of hemorrhage postoperatively (85% of the included patient were CT scanned after insertion). The authors state that no infections were diagnosed in the study population; however, one patient had a fever and a positive bacterial culture from the catheter tip, but no bacterial growth in the CSF. A large study by Koskinen and Olivecrona [78] state, that after insertion of almost 1000 Codman MicroSensors, only 3 incidents of surgically related hemorrhages were found, none of which required surgical intervention. No infections were linked to the placement of the MicroSensor.

Regarding the Raumedic Neurovent-P Sensors, Citerio et al. [79] tested a total of 99 sensors in an equal number of patients. No CNS infections were registered. 2 patients had smaller hemorrhages, not requiring intervention.

The relatively new Pressio sensor has yet to be tested thoroughly *in vivo*. Only clinical study currently available, is that by Lescot et al. [80], looking at the accuracy of 15 Pressio sensors and 15 Codman MicroSensors in comparison to ICP measurements undertaken by an EVD. The two types of sensors performed much alike, albeit with a mean difference of ±7 mmHg compared to ICP values acquired by the EVDs. Complications were not recorded. 

The Spiegelberg sensor has been tested by Lang et al. [81], without any incidence of hemorrhage among all 87 patients (all patients were CT-scanned after insertion). None of the patients showed clinical signs of meningitis. In three patients, a leak related to the sensor resulted in incorrect measurements. Kiening et al. [82] additionally tested the Spiegelberg sensor for use in continuous intracranial compliance (cICC) monitoring in ten patients with TBI and found poor overall cICC data quality as well as poor predictive capabilities in identifying increased ICP and correlation with cerebral hypoxia. Despite unsatisfactory results in the current clinical care setting, the same authors [83] later found a correlation between increasing age and decreasing compliance and speculated that this correlation might contribute to the poorer outcome seen in elderly patients after TBI.

It is worth mentioning, that the Neurovent-P sensor, the Spiegelberg sensor and the Codman MicroSensor are compatible with magnetic resonance imaging (MRI) without any danger to the patient. The Camino monitor and Pressio sensor contain ferromagnetic components, and therefore patients with these devices cannot undergo MRI [84–86].

Generally, several of the above studies concluded that when it comes to measuring ICP, microtransducers are just as accurate as the EVDs [69, 81]. However, microtransducers share a common disadvantage, in that no recalibration is possible after placement; though the Spiegelberg catheter is an exception from this rule, as it recalibrates itself every hour. The EVDs, on other hand, have the advantage of that they can be recalibrated at any time, simply by resetting the transducer to atmospheric pressure at the level of the so called zero reference point (Foramen of Monro/Tragus). The amount of CSF drained depends on the pressure gradient inside the CSF cavity and the resistance of the CSF drainage, the resistance being defined by the pressure gradient of the CSF has to overcome to reach the level of the drip chamber. This means that the position of the drip chamber relative to the CSF space is the crucial factor for the amount of drained CSF. When recording ICP, it is also of utmost importance that the “three-way” valve system is closed to drainage, so that the true ICP is measured, otherwise it will be the drainage pressure that is recorded. 

Problems often arise due to incorrect setup of the system, mainly resulting in a false pressure gradient and secondarily problems in insufficient CSF drainage, as well as false ICP measurements du to incorrect use of the “three-way” valve.

The lack of continuous calibration can cause the sensor to report imprecise ICP values. The difference between the starting ICP value when the sensor is calibrated (0 mmHg), and the ICP value that is measured when the sensor is removed is termed “zero drift.” A large difference between these two ICP measurements indicates, that the ICP measured while the device was implanted in the patient, was not the “true” ICP at any given moment. Therefore, the cumulative pressure difference can have important implications for the treatment and prognosis of the patient. Data regarding differences between microtransducer ICP monitoring devices is summarized in Table 2.

## 3. Noninvasive Methods of Measuring

The idea of a noninvasive method of measuring ICP is captivating, as complications seen in relation to the invasive methods of ICP measuring, that is, hemorrhage and infection, are avoidable. Different techniques have been proposed; however, in this paper we will focus on the ones most widely familiar.

### 3.1. Transcranial Doppler Ultrasonography (TCD)

The TCD technique applies ultrasound to initially measure the blood flow velocity in the middle cerebral artery. The difference between systolic and diastolic flow velocity, divided by the mean flow velocity, is called the pulsatility index (PI):

(1) $$\text{PI}=\frac{\text{systolic}\;\text{flow}\;\text{velocity}-\text{diastolic}\;\text{flow}\;\text{velocity}}{\text{mean}\;\text{flow}\;\text{velocity}}.$$

PI is found to correlate with invasively measured ICP [90–92], and correlation coefficients between 0,439 and 0,938 have been found. Bellner et al. [90] reported the best correlation and a mean deviation of ±4,2 mmHg from invasively measured ICP. A deviation of this small magnitude is clinically acceptable. However, this small magnitude of deviation only applies to ICP values lower than approx. 30 mmHg. At higher ICP values, the magnitude of deviation increases, making accurate ICP measurements impossible. The apparently high correlation includes great individual variations in the data. It is seen that a PI of 1 can mean anything from a few mmHg to about 40 mmHg. A variation of this magnitude is clearly unacceptable for clinical use. 

Behrens et al. [93] described a similar large spread in their experiment, where 10 patients with idiopathic normal pressure hydrocephalus had their ICP artificially heightened or lowered by lumbar infusion. ICP calculated from PI was compared to ICP measured by a Codman MicroSensor. They reported that an ICP of 20 mmHg found by using PI had 95% confidence intervals of −3,8 to 43,8 mmHg. Brandi et al. [94] also came to the conclusion that PI-calculated ICP is too uncertain. In their study, 45 sedated patients with severe traumatic brain injury, each monitored with a Raumedic probe, were examined daily using TCD. Their PI and calculated ICP were compared to the invasively found ICP. The best correlation was found by using the calculations proposed by Bellner et al. [90], which yielded a mean difference of −3,2 ± 12,6 mmHg.

Apart from being imprecise, the technique requires training and repetitive exercise [95], and there is also intra- and inter-observer variations as noted earlier [95–98]. Furthermore, the technique cannot be used on 10–15% of the patients due to the ultrasound not being able to penetrate the skull (the so-called bone window) [99].

### 3.2. Tympanic Membrane Displacement (TMD)

The technique takes advantage of the communication of the CSF and the perilymph via the perilymphatic duct. Stimulation of the stapedial reflex causes a movement of the tympanic membrane, which is shown to correlate to ICP [100, 101]. Stapes rests on the oval window, which is covered by a membrane. This membrane is flexible, meaning that the pressure of the fluid in the cochlea affects how the membrane and stapes are positioned and how they move. A quantitative measurement of this movement is the fundament of this technique. However, the technique is not without flaws; Shimbles et al. [102] tested the technique on 148 patients with intracranial pathology (hydrocephalus and benign intracranial hypertension) and on 77 healthy controls. The technique was applied successfully on 70% of the healthy subjects, but only on 40% in the patient population. It was noted, that the low rate of success was mainly due to the perilymphatic duct being less passable with age, especially after the age of 40. 

Furthermore, a subgroup of cases in the patient population were invasively ICP monitored at the time of the experiment. A correlation between the invasively and the TMD measured ICP values were found. However, intersubject variability was so great that the predictive limits of the regression analysis was an order of magnitude greater than normal ICP range, thus precluding the method for clinical use [102].

### 3.3. Optic Nerve Sheath Diameter (ONSD)

The optic nerve is part of the central nervous system, and therefore surrounded by the dural sheath. Between the sheath and the white matter is a small 0,1-0,2 mm subarachnoid space, which communicates with the subarachnoid space surrounding the brain. In cases of increased ICP, the sheath expands. Changes in the diameter of the nerve sheath can be visualized using transocular ultrasound. Several studies [103–106] have investigated the correlation between the nerve sheath diameter and invasively measured ICP. A correlation coefficient of between 0,59–0,73 has been found. The technique is cheap and efficient; the examination takes around 5 minutes per patient [104]. However, as with all ultrasonography, it requires training and has intra- and inter-observer variance, though these variations are minor. Mean intraobserver variance ± 0,1-0,2 mm and mean interobserver variance ± 0,2-0,3 mm was found in recent studies [105, 107, 108]. Furthermore, it is important to mention that several conditions can affect optic nerve diameter, for example, tumors, inflammation, Graves disease, and sarcoidosis [105, 109]. Patients with glaucoma and cataract have been excluded from the above study population, and it is therefore uncertain, whether the technique can be applied on patients with these common conditions. Lesions of the orbita or the optic nerve are present in 10% of all head trauma cases, which can render measurements with the ONSD impossible.

At present, the technique does not seem to be accurate enough to be used as a replacement for invasive ICP measuring methods. It can, however, distinguish between normal and increased (>20 mmHg) ICP. A review article [109] showed that all included studies found a cutoff value of 5,00–5,90 mm for predicting increased ICP. Sensitivity was 74–95% and specificity 79–100%. Another study by Rajajee et al. [106] recently published even better results, with sensitivity of 96% and a specificity of 94% for increased ICP (>20 mmHg) for a ONSD cutoff value of 4,8 mm. This means that this technique can potentially be used as a screening method for detecting raised ICP in settings, where invasive ICP monitoring capabilities are not available, that is, hospitals without access to a neurosurgeon.

### 3.4. Magnetic Resonance Imaging (MRI) & Computer Tomography (CT)

In 2000, Alperin et al. investigated the possibilities of using MRI as a noninvasive method of ICP measurement [110]. By using motion-sensitive MRI, pulsatile arterial, venous, and CSF flow in and out of the cranial vault during the cardiac cycle was measured. A small volume change (about 1 mL) during the cardiac cycle was found and calculated from the net transcranial CSF and blood volumetric flow rates, and the following change in pressure was estimated from CSF velocity. An elastance index was derived from the ratio of pressure to volume change and found to correlate well with invasively measured ICP (*R* ^2^ = 0,965; *P* < 0,005). However, as Marshall and colleagues pointed out, care is required in the selection of representative image slides as well as choosing the representative blood vessels [111]. Furthermore, the technique is very sensitive to differences in heart rate measured in the circulation contra the CSF flow rate as well as CSF measurements. Even when the above was addressed, some subjects displayed significant variation between repeated measurements, requiring for the data gathered from individual cases to be interpreted with caution [111]. However, if we can accept these shortcomings, the technique could have a role as a screening tool for identification of patients in need of invasive ICP monitoring after moderate head trauma. It could also play a role in diagnosis and evaluation of several chronic disorders potentially associated with increased ICP values, i.e., hydrocephalus, pseudotumor cerebri, intracranial mass lesions, and so forth [112].

A way of interpreting ICP values from cranial CT scans has also been investigated; the majority of studies were conducted in the late 1980s and early 1990s and failed to show consistent correlation between ICP and CT scan characteristics [113]. In 2003, Eide reported no significant correlation between actual size (or change in size) of cerebral ventricles by cranial CT scans and invasively monitored ICP in 184 consecutive patients [114]. A linear, but ultimately nonpredictive relationship between baseline ICP and initial head CT scan characteristics was found by Miller and colleagues [113]. Similar results were observed by Hiler et al. who concluded, after looking at 126 patients with severe traumatic brain injury, that mean ICP values in the first 24 hours cannot be predicted by using the Marshall CT scan classification [115]. Overall, no method of estimating ICP on the basis of cranial CT scans currently exists.

### 3.5. Fundoscopy and Papilledema

Papilledema, or optic disc swelling, due to raised ICP can be visualized by fundoscopy and graded by the Frisén Scale into 5 categories depending on signs of disturbed axoplasmic transport. A study employing fundus photographs showed good reproducibility of this grading scale among different observers, specificity ranging between 88–96% and sensitivity between 93–100% [116]. Nevertheless, even though fundoscopy is often used as a screening method in cases of suspected increase in ICP, the grading scale is not widely applicable or accepted. The technique itself is limited to the abilities of the examiner, as well as the circumstances surrounding the examination, the examiner requiring good visualization of the optic disc to be able to detect papilledema [117].

Furthermore, since the process of optic disc swelling in cases of raised ICP takes time, the technique cannot be applied in emergency situations with sudden increases in ICP, such as, trauma [118].

## 4. Discussion

ICP monitoring techniques are multiple and diverse. Nevertheless, before choosing the technique to apply in critical care, several factors need to be considered; the precision of measurements made, the cost of the device as well as the possible complications and mechanical problems associated with the individual techniques.

In regards to precision of measuring accurate ICP values, EVDs are considered the gold standard, closely followed by microtransducers, which measure ICP almost just as accurately [69, 81]. The noninvasive techniques have their greatest shortcomings in this field. At present, none of the above-mentioned noninvasive techniques are accurate enough to be used in a critical (intensive) care setting.

On the other hand, the noninvasive techniques have their advantages in completely avoiding complications such as hemorrhages and infections, which are often associated with the invasive techniques.

Clinically relevant hemorrhaging, that is, those causing neurological deficits or requiring surgical intervention occur in about 0,5% of cases with EVDs [51, 52], and approximately the same percentage applies for microtransducer techniques [74, 76–78, 81]. A clinically relevant hemorrhage percentage of 0,5% associated to the invasive techniques may not seem as much, but one has to keep in mind that this means one in 200 patients will have a worsened clinical outcome solely due to the application of an invasive ICP monitoring technique. This is an intensely debated issue, especially given the fact that general guidelines for ICP monitoring are not widely accepted, resulting in variations for application of invasive ICP monitoring among hospitals [8, 22, 46]. One could fear that a too liberal approach for invasive ICP monitoring could result in unnecessary worsened clinical patient outcome, without the monitoring itself having any relevance to the way these patients are treated.

The same words of caution can then applied in relation to the relatively high postoperative infection frequencies of up to 27% [53] in relation to the insertion of an EVD. A significant number of these patients will develop systemic or cerebral infections, with subsequent risk of increased mortality and morbidity, for example, hydrocephalus, infarcts, epilepsy, or cranial nerve palsy [119]. As Dasic et al. [57] showed, the only way to minimize the risk for postoperative infection is to strictly follow sterile guidelines. It is also worth mentioning that patients with microtransducers generally have a lower rate of postoperative infections than patients with EVDs, ranging between 0–8,5% [74, 76–78, 81].

The rate of nonfunctioning EVDs has been found to be 6,3% [69]. One would like to think that this relative simple technology would be more reliable than the more complex microtransducers. However, microtransducers are only defective in 3,14–5,0% of cases [74–76, 81]. In most part, the relatively high percentage of malfunctioning EVDs is due to the EVD being placed intraparenchymaly or blocked with pieces of brain matter and blood clots [67–69].

For the reviewed noninvasive techniques, there are several patient categories for which the measuring technique cannot be applied in critical care practice. In 10–15% of patients investigated using TCD, no valid measurements could be made [99]. In measurements using TMD, the figure was 60% [102], and for ONSD 10% [103, 105].

The cost for placing an EVD amounts to around $200 in materials [45, 69]. Microtransducers are more expensive, given that they require a monitor, with the total cost easily around several thousand dollars, and the transducers themselves costing at least $400–600 each [45, 69]. In addition to the equipment comes also the subsequent cost of maintaining and replacing it. The noninvasive techniques require only the single expense of purchasing the device, after which the devices can be applied multiple times without further costs apart from wages and maintenance.

To summarize, economically and complication wise, the noninvasive techniques are favored. However, considering the high number of patients where noninvasive techniques cannot be applied, and more importantly, the low accuracy of the ICP measurements undertaken, the noninvasive techniques are less favorable. The current noninvasive techniques are simply not accurate enough to replace the traditional invasive techniques. This leaves us with the choice between EVDs and microtransducers. Precision wise, there is not a great difference between these two techniques, despite the fact that most microtransducers cannot be recalibrated. However, in this context it is important to point out that the Camino MicroSensor has problems with a large zero drift [74, 75]. Economically, microtransducers are more expensive, but apparently they carry a lower rate of postoperative infections. EVDs, on the other hand, have the advantage that they can be used for drainage of CSF and administering of drugs intrathecally. Drainage of CSF has been used in routine clinical practice for lowering of ICP. However, this has not been shown to better the cerebral perfusion of the patient nor improve the patients' final clinical outcome [120].  Table 3 summarizes the results. 

The American Brain Trauma Foundation still has EVDs as their favored method of ICP monitoring [69]. Whether EVD or microtransducers is the optimal ICP measuring technique is a difficult question to answer unambiguously, as both have advantages and disadvantages as discussed above. Both can, therefore, be regarded as good options when ICP monitoring is needed.

But when is ICP monitoring needed in critical care? As mentioned in the background paragraph, there are no general guidelines. A thorough evaluation of the literature is outside the scope of this paper, but we will comment briefly on the topic. Stein et al. [121] did an extensive review on all available articles on outcome of severe traumatic brain injury from the last forty or so years, resulting in 127 case series, involving more than 125.000 patients. Overall, higher-intensity treatment with use of ICP monitoring resulted in a 12% lower mortality rate and a 6% better chance of favorable outcome compared to less aggressive treatment and monitoring approach, where ICP monitoring was not applied. However, most of the reviewed studies are of a methodologically limited quality. A recent Cochrane review [22] also sought to review the literature, but all articles that were obtained had to be excluded since none were of a prospective randomized nature and therefore not deemed as “sufficient” evidence.

A large part of the evidence advocating ICP monitoring originates from the late nineteen seventies and early eighties [122–124], but these studies are of weaker methodological design. Current studies reporting better survival and outcome with ICP monitoring include Patel et al. [125] and Fakhry et al. [126]. However, several other studies have reached the opposite conclusion, indicating worse outcome as a consequence of ICP monitoring and CPP-oriented therapy [46, 127–129].

Nevertheless, to this date, no prospective randomized study of the possible benefits of ICP monitoring has been done. Current ICP- and CPP-based therapies are based on differing assumptions of “elevated pressure,” with recommendation on treatment initiation at ICP levels above 20–25 mmHg [17]. It is apparent today that additional neuromonitoring modalities should supplement ICP in the critical care setting, thereby increasing patient safety by more accurately guiding treatment interventions in terms of type, aggressiveness and duration, including controlled tapering [130].

## 5. Conclusion

This paper sought (1) to provide an overview of “pros” and “cons” of the most widely used methods of ICP monitoring and (2) to evaluate whether noninvasive techniques could be used as an alternative to the invasive techniques in critical care.

To answer the first question, we can conclude that both EVD and microtransducers are good technologies for ICP monitoring. Both are accurate in ICP monitoring, but have risks of complications in the form of postoperative hemorrhage and infection. Which of these modalities is preferable, must ultimately be decided by the individual clinician and department.

To answer the second question, we conclude that noninvasive techniques lack the accuracy of their invasive counterparts. Additionally, the noninvasive ICP monitoring cannot be carried out on a large percentage of patients due to anatomical variations, leading us to conclude that current noninvasive techniques cannot be used as an alternative to the invasive techniques.

## Figures and Tables

**Table 1:** Conditions where ICP-monitoring is used.

Traumatic head injury Intracerebral hemorrhage Subarachnoid hemorrhage Hydrocephalus Malignant infarction Cerebral edema CNS infections Hepatic encephalopathy	

Modified from Smith [8].

**Table 2:** Comparison of microtransducer ICP monitoring devices.

	Technology	Rate of infection	Rate of hemorrhaging	Technical errors	Zero drift
Camino ICP Monitor	Fiber optic	8,5% [74]	2,50% (0,66% clinical significant) [74]	4,5% [74]	Mean 7,3 ± 5,1 mmHg (range −17 to 21 mmHg) [74]
		4,75% [76]	1,1% [76]	10% [75]	Mean −0,67 mmHg (range −13 to 22 mmHg) [75]
				3,14% [76]	Mean 3,5 ± 3,1 mmHg (range 0 to 12 mmHg) [84]

Codman MicroSensor	Strain gauge	0% [77]	0% [76]	n/a	Mean 0,9 ± 0,2 mmHg (range −5 to 4 mmHg) [78]
		0% [78]	~0,3% (0% clinical significant) [78]		Mean 0,1 ± 1,6 mmHg/100 hours of monitoring [80]
					Mean 2,0 mmHg (range −6 to 15 mmHg) [87]

Raumedic Neurovent-P ICP sensor	Strain gauge	0% [79]	2,02% (0% clinical significant) [79]	n/a	Mean 0,8 ± 2,2 mmHg (range −4 to +8 mmHg) [79]
					1,7 ± 1,36 mmHg (range −2 to 3 mmHg) [84]
					*In vitro:* 0,6 ± 0,96 mmHg (range 0 to 2 mmHg) [88]

Pressio	Strain gauge	n/a	n/a	n/a	Mean −0,7 ± 1,6 mmHg/100 hours of monitoring [80]
					*In vitro:* 7-day drift <0,05 mmHg [89]

Spiegelberg	Pneumatic	0% [81]	0% [81]	3,45% [81]	Mean < ± 2 mmHg [81]

**Table 3:** The different technologies compared.

Technology	Accuracy	Rate of infection	Rate of hemorrhaging	Cost per patient	Miscellaneous
External ventricular drainage	High	Low to moderate	Low	Relatively low	Can be used for drainage of CSF and infusion of antibiotics
Microtransducer ICP monitoring devices	High	Low	Low	High	Some transducers have problems with high zero drift
Transcranial Doppler ultrasonography	Low	None	None	Low	High percentage of unsuccessful measurements
Tympanic membrane displacement	Low	None	None	Low	High percentage of unsuccessful measurements
Optic nerve Sheath diameter	Low	None	None	Low	Can potentially be used as a screening method of detecting raised ICP
MRI/CT	Low	None	None	Low	MRI has potential for being used for noninvasive estimation of ICP
Fundoscopy (papilledema)	Low	None	None	Low	Can be used as a screening method of detecting raised ICP, but not in cases of sudden raise in ICP, that is, trauma
